# Neuromodulation Therapy with Vagus Nerve Stimulation for Intractable Epilepsy: A 2-Year Efficacy Analysis Study in Patients under 12 Years of Age

**DOI:** 10.1155/2016/9709056

**Published:** 2016-02-10

**Authors:** Suresh Gurbani, Sirichai Chayasirisobhon, Leslie Cahan, SooHo Choi, Bruce Enos, Jane Hwang, Meei Lin, Jeffrey Schweitzer

**Affiliations:** ^1^Comprehensive Epilepsy Program, Southern California Permanente Medical Group, CA, USA; ^2^Department of Neurology, Kaiser Permanente Medical Center, Suite No. 208, 3460 E. La Palma Avenue, Anaheim, CA 92806, USA

## Abstract

To study the efficacy of vagus nerve stimulation (VNS) therapy as an adjunctive treatment for intractable epilepsy in patients under 12 years of age, we analyzed 2-year postimplant data of 35 consecutive patients. Of the 35 patients, 18 (51.4%) at 6 months, 18 (51.4%) at 12 months, and 21 (60.1%) at 24 months showed ≥50% reduction in seizure frequency (responders). Although incremental seizure freedom was noted, no patient remained seizure-free throughout the 3 study periods. Partial response (≥50% seizure reduction in 2 or less study periods) was seen in 8 (22.9%) patients. Twelve patients (34.3%) were nonresponders. Out of 29 patients with primary generalized epilepsy, 20 (68.9%) and, out of 6 patients with focal epilepsy, 3 (50%) had ≥50% seizure control in at least one study period. No major complications or side effects requiring discontinuation of VNS therapy were encountered. We conclude that (1) patients with intractable primary generalized epilepsy respond better to VNS therapy, (2) cumulative effect of neuromodulation with improving responder rate to seizure freedom with continuation of VNS therapy is noted, and (3) VNS therapy is safe and is well tolerated in children receiving implant under 12 years of age.

## 1. Introduction

Neuromodulation therapies are nonpharmacotherapeutic options for patients with drug resistant epilepsy who are not candidates for resective epilepsy surgery. In 1997, the US Food and Drug Administration (FDA) approved vagus nerve stimulation (VNS) implant as adjunctive therapy for reducing the frequency of seizures in patients >12 years of age with partial-onset seizures refractory to antiepileptic drugs (AEDs) [1].

Initial studies with randomized controlled trials reporting on the efficacy of VNS involved rather short follow-up duration (3 months to 3.5 months) and ≥50% seizure reduction ranged from 23.4% to 39% of the patients [2–5]. Since 1999, several studies have reported long-term follow-up ranging from 6 months to 10 years with ≥50% seizure reduction observed in 35% to 63.8% of the patients [6–22].

Literature search identified 16 studies regarding the efficacy of VNS in children [17, 18, 23–36]. However, most of these studies did not truly reflect the efficacy of VNS therapy in children <12 years old as they included older patients as well. Ten of the 16 studies included subjects through 18 years of age, and one each included subjects up to 19, 20, 21, and 25 years of age. One study of 11 patients with tuberous sclerosis had a mean age of 14 years with a range from 2 to 35 years [23]. In this study, we report long-term (2 years) observation on the efficacy and the safety of VNS in epileptic patients <12 years of age.

## 2. Methods

Ours is a prospectively collected data analysis retrospective study. All patients with epilepsy being treated at our center maintain daily seizure diary which is entered in the electronic database during follow-up visits. All patients with recalcitrant epilepsy undergo long-term video EEG monitoring and neuroimaging studies including MRI brain examination and are presented at Kaiser multidisciplinary epilepsy surgery case conference to discuss alternate nonpharmacotherapeutic treatment options. A total of 160 patients with drug resistant epilepsy (failed at least 3 AEDs at adequate doses appropriate for the type of epilepsy) who were not candidates for resective surgery received VNS implant from September 1998 to December 2011, 35 of whom were <12 years of age at the time of implant. VNS device from Cyberonics was implanted by our neurosurgeons who had received the required training.

To allow wound healing, the VNS implant was not activated until one week postoperatively. Output current was gradually increased in 0.25 mA increments once per week at six weekly visits, then at six subsequent biweekly visits, and then at each of three monthly visits to the clinic. After an informed decision by the parents, type of VNS cycling (standard versus rapid) and parameters were selected by the treating pediatric epileptologist. For standard cycling, the signal on-time was ≥30 seconds and signal off-time ranged from 3 to 5 minutes. For rapid cycling, signal on-time was ≤21 seconds and signal off-time ranged from 0.2 to 1.8 minutes. Rapid cycling was initiated with the parameters of 7 seconds on-time and 0.2 minutes off-time. Output current was adjusted depending on the patient's tolerance to the electrical stimulation and seizure control. Maximum output current used was 3.0 mA for rapid cycling and 3.5 mA for standard cycling. Ongoing AED regimen (dosing regimen and if needed AED) was adjusted as clinically warranted.

The efficacy of VNS therapy was analyzed by comparing the mean seizure frequency (prior 8-week period) at baseline (at VNS implant) to that at 6-month, 12-month, and 24-month postimplant study points. We defined the efficacy of VNS therapy as follows: (1) responders: ≥50% reduction of seizures in all three study periods, (2) partial responders: ≥50% reduction of seizures in 2 or less study periods, and (3) nonresponders: <50% response and/or worsening seizure control in all 3 study periods. Efficacy of standard versus rapid cycling therapy parameters was also studied. We analyzed the efficacy of VNS therapy according to the type of epilepsies as well. We also assessed for postoperative adverse events, side effects, and tolerability of both the surgical implantation procedure and the VNS device.

## 3. Results

**Figure 1:**
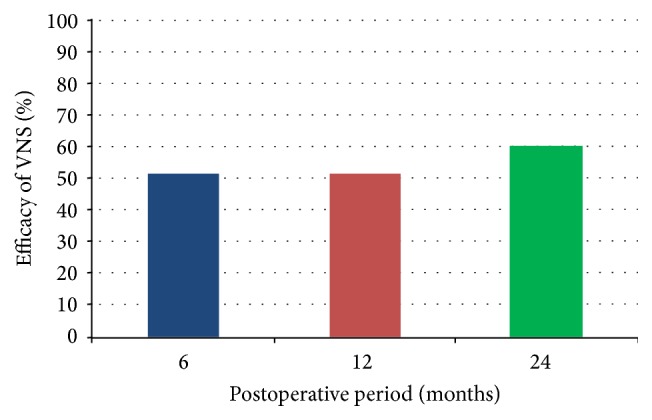
Efficacy of VNS (≥50% reduction in seizure frequency) at 3 study periods.

Thirty-five patients (23 males, 12 females) with age ranging from 5 years to 12 years (mean age, 7.79 ± 2.65 years) met the selection criteria. Clinical characteristics for the patients at baseline are summarized in Table 1. Mean age of onset of epilepsy was 1.25 ± 1.55 years. Mean duration of epilepsy before the VNS implant was 6.67 ± 2.95 years. Mean number of AEDs at baseline was 2.5. Mean output current setting was 1.9 ± 0.7 mA at 6 months, 2.3 ± 0.7 mA at 12 months, and 2.5 ± 0.7 mA at 24 months. Of the 35 patients, 18 (51.4%) at 6 months, 18 (51.4%) at 12 months, and 21 (60.1%) at 24 months showed ≥50% reduction in seizure frequency (Figure 1).

Although, among the responders, a complete (100%) seizure control was seen in 4 of 18 patients (22.2%) at 6-month, 5 of 18 patients (27.8%) at 12-month, and 7 of 21 patients (33.3%) at 24-month follow-up period, no single patient remained seizure-free throughout the 3 study periods. A total of 15 (42.9%) patients had ≥50% reduction in seizure frequency in all three periods and a partial response was seen in 8 (22.8%) more patients. Twelve patients (34.3%) showed no clinically significant benefit in all three periods (Table 2).

For the 22 patients who were treated with rapid cycle of VNS, the output current ranged from 0.75 mA to 3 mA and pulse width from 125 microseconds to 250 microseconds, with signal on-time set at 7 seconds to 14 seconds and signal off-time ranging from 0.5 minutes to 1.1 minutes. Seizure frequency reduction of ≥50% was seen in 11 of 22 patients (50%) at 6-month, 11 of 22 patients (50%) at 12-month, and 12 of 22 patients (54.5%) at 24-month follow-up period. The remaining 13 patients were treated with the standard cycle of VNS with output current ranging from 0.75 mA to 3.5 mA and pulse width from 250 microseconds to 500 microseconds, with signal on-time of 30 seconds and signal off-time from 3 minutes to 5 minutes. Seizure frequency reduction of ≥50% was seen in 7 of 13 patients (53.8%) at 6-month, 6 of 13 patients (46.2%) at 12-month, and 9 of 13 patients (69.2%) at 24-month follow-up period.

The efficacy of VNS according to the etiologies of epilepsy is shown in Table 3 and according to the seizure types in Table 4. During the study period 25 patients had a single type of seizures, 6 patients had 2 types of seizures, and 4 patients had 3 types of seizures. Out of 35 patients, 29 (82.9%) had primary generalized epilepsy and 6 (17.1%) had focal epilepsy. Twenty patients (68.9%) with primary generalized epilepsy and 3 patients (50%) with focal epilepsy had ≥50% reduction in seizure frequency. Best responders were patients with primary generalized epilepsy with tonic-clonic seizures followed by primary generalized epilepsy with atypical absence seizures.

Cough and pharyngeal paresthesia commonly occurred during initial application or ramming up of output current. These adverse events were successfully managed by adjusting the parameters. No side effects necessitating discontinuation of VNS therapy were encountered.

## 4. Discussion

VNS therapy has been approved as adjunctive treatment for drug resistant focal epilepsy in patients >12 years of age [1]. However, as drug resistant epilepsies in pediatric population are also an unconquered challenge despite availability of second and third generation AEDs and as the epilepsy treatment goal remains seizure freedom, VNS therapy has been used in patients <12 years of age as well. Many researchers have reported the efficacy of VNS for treatment of epilepsy in children [17, 18, 23–36]. Among these 16 studies, ten studies included subjects through 18 years of age, and one each included subjects up to 19, 20, 21, and 25 years of age. One study of 11 patients with tuberous sclerosis had a mean age of 14 years with a range from 2 to 35 years [23]. Our study reports the efficacy and safety of VNS therapy in a group of 35 epileptic patients <12 years of age.

Similar to the observations of prior VNS studies, with increasing duration of VNS therapy, a trend towards improving responder rate and seizure freedom was noted in our study as well [37–39]. The exact mechanism for the improving efficacy of VNS with increasing duration of therapy is not fully understood. Chronic therapeutic response to VNS therapy is highly correlated with bilateral thalamic increases in synaptic activity [40]. During chronic VNS therapy, brain excitatory amino acid neurotransmitter levels are reduced and inhibitory neurotransmitter levels are increased but no direct relationship to seizure control has been noted [40].

Optimal parameter settings for VNS therapy are not yet well defined. In current study, both rapid and standard cycle settings of VNS parameters were used. Patients who were treated with rapid cycling showed ≥50% reduction of seizure frequency in all three periods while those treated with standard cycling showed ≥50% reduction of seizure at 6-month and 24-month periods. Both rapid cycling and standard cycling demonstrated the cumulative seizure response to VNS therapy at 24-month period. The comparison of the efficacy between rapid cycling and standard cycling was not conclusive. Our previous study in 39 patients (age ranging from 5 to 72 years) demonstrated a trend towards greater seizure frequency reduction in patients with rapid cycle than standard cycle parameters. It also showed that when compared to adult patients, the response to rapid cycle in pediatric patients was greater [41]. However, a 2-year follow-up study has reported greater overall seizure frequency reduction with the standard cycle than the rapid cycle [12]. Other studies did not show any difference in responder rate with either cycle [24, 29]. More research with larger population is recommended to study this further.

In our study, VNS therapy was effective in both focal epilepsy and some types of generalized epilepsy. VNS therapy has been reported to be effective in patients with Lennox-Gastaut syndrome [27, 29, 30] and tuberous sclerosis complex [36, 37]. In our study, VNS therapy was effective in achieving ≥50% reduction in seizure in patients with Lennox-Gastaut syndrome, encephalitis, cortical dysgenesis, perinatal encephalopathy, and tuberous sclerosis complex. Best responders were patients with primary generalized epilepsy with tonic-clonic seizures followed by primary generalized epilepsy with atypical absence seizures. Small sample size did not permit statistical analysis by seizure types and etiologies.

Our patients tolerated the VNS implantation well. There was no serious wound infection requiring explantation. No major complications or side effects requiring discontinuation of VNS therapy occurred during the 2-year study period.

Patients included in this study were offered VNS as an adjunctive treatment modality to ongoing antiepilepsy medication regimen and not after failing most available antiepilepsy medications. Therefore, change in the dosing regimen and, if needed, in the antiepilepsy medications used (which is a limitation of this study) was done per the choice of the treating physician. As many more AED choices (second and third generation AEDs) are available now, with a small study subject size it was not possible to comment on synergism of any specific AED mechanism of action being responsible for the seizure control noted after VNS implant.

Many studies have reported lack of improvement in responder rate after failing 2 appropriate AEDs [1]. All the patients in the current study had failed at least 3 AEDs in adequate doses appropriate for the type of epilepsy before VNS therapy was initiated. Therefore, it can be safely assumed that in these patients maximum response to AEDs had already been attained and reduction in seizure frequency noted after the implant can be attributed to the VNS therapy. On the other hand, the patient's desire to decrease the dose and reduce the number of AEDs after VNS implantation may have resulted in a negative impact on the responder rate.

In conclusion, neuromodulation with VNS therapy can be used successfully as an adjunctive treatment for patients <12 years of age with both focal and generalized drug resistant epilepsies.

The cumulative seizure response to VNS therapy necessitates long-term efficacy analysis studies. Due to availability of third generation AEDs it is unethical to design a prospective double blind research study with unchanged AED regimen to further define the findings of this study. Therefore, our retrospective data analysis study has limited but definite scientific contribution.

## Figures and Tables

**Table 1:** Clinical data of patients at baseline.

(A) Epilepsy and seizure types	
Generalized tonic-clonic	9 patients
Absence atypical	5 patients
Tonic	3 patients
Myoclonic	1 patient
Atonic	1 patient
Focal with secondary generalized tonic-clonic	4 patients
Focal with dyscognitive features	2 patients
Mixed	10 patients
(B) Etiology	
Lennox-Gastaut syndrome	13 patients
Postencephalitis	5 patients
Cortical dysgenesis	4 patients
Postanoxic encephalopathy	3 patients
Idiopathic	3 patients
Tuberous sclerosis	3 patients
Chromosomal abnormality	2 patients
Stroke	2 patients

**Table 2:** The efficacy of VNS with ≥50% reduction of seizures for all patients at 6 months, 12 months, and 24 months.

Study period	Number of patients	Percent
At 6 months, 12 months, and 24 months	15	42.9
At 6 months	2	5.7
At 6 months and 24 months	1	2.8
At 12 months and 24 months	3	8.6
At 24 months	2	5.7
No response	12	34.3

**Table 3:** The efficacy of VNS with ≥50% reduction of seizures according to the etiologies of epilepsy.

Etiology (patients)	Responders	Partial responders	Nonresponders
Lennox-Gastaut syndrome (13)	38.5%	23.0%	38.5%
Postencephalitis (5)	20.0%	40.0%	40.0%
Cortical dysgenesis (4)	75.0%	0%	25.0%
Postanoxic encephalopathy (3)	33.3%	0%	66.7%
Perinatal encephalopathy (3)	66.7%	0%	33.3%
Tuberous sclerosis (3)	50.0%	50.0%	0%
Chromosomal abnormality (2)	50.0%	0%	50.0%
Stroke (2)	50.0%	50.0%	0%

**Table 4:** Number of patients with ≥50% reduction of seizures according to the seizure types during 3 study periods.

Seizure type (# at baseline)	6 months	12 months	24 months
Generalized tonic-clonic (16)	10	11	11
Absence atypical (8)	6	5	5
Tonic (8)	4	4	4
Myoclonic (4)	3	1	0
Atonic (5)	2	3	2
Focal with generalized tonic-clonic (4)	0	2	2
Focal with dyscognitive features (2)	1	1	1
